# The Native Arbuscular Mycorrhizal Fungi and Vermicompost-Based Organic Amendments Enhance Soil Fertility, Growth Performance, and the Drought Stress Tolerance of Quinoa

**DOI:** 10.3390/plants11030393

**Published:** 2022-01-31

**Authors:** Wissal Benaffari, Abderrahim Boutasknit, Mohamed Anli, Mohamed Ait-El-Mokhtar, Youssef Ait-Rahou, Raja Ben-Laouane, Hela Ben Ahmed, Toshiaki Mitsui, Marouane Baslam, Abdelilah Meddich

**Affiliations:** 1 Center of Agrobiotechnology and Bioengineering, Research Unit labelled CNRST (Centre AgroBiotech-URL-CNRST-05), “Physiology of Abiotic Stresses” Team, Cadi Ayyad University, Marrakesh 40000, Morocco; wissalbenaffari1@gmail.com (W.B.); Abderrahim.boutasknit@gmail.com (A.B.); moh1992anli@gmail.com (M.A.); youssefaitrahou41@gmail.com (Y.A.-R.); benlaouaneraja@gmail.com (R.B.-L.); 2 Laboratory of Agro-Food, Biotechnologies and Valorization of Plant Bioresources (AGROBIOVAL), Faculty of Science Semlalia, Cadi Ayyad University, Marrakesh 40000, Morocco; mohamed.aitelmokhtar@gmail.com; 3 Laboratoire Mixte Tuniso-Marocain (LMTM) de Physiologie et Biotechnologie Végétales et Changements Climatiques LPBV2C, Tunis 1000, Tunisia; hela.benahmed@fst.utm.tn; 4 Laboratory of Biochemistry, Faculty of Agriculture, Niigata University, Niigata 950-2181, Japan; t.mitsui@agr.niigata-u.ac.jp

**Keywords:** biofertilizers, nutrient acquisition, crop sustainability, plant metabolism, plant-microbe interactions, water scarcity

## Abstract

The present study aimed to determine the effects of biostimulants on the physicochemical parameters of the agricultural soil of quinoa under two water regimes and to understand the mode of action of the biostimulants on quinoa for drought adaptation. We investigated the impact of two doses of vermicompost (5 and 10 t/ha) and arbuscular mycorrhizal fungi applied individually, or in joint application, on attenuating the negative impacts of water shortage and improving the agro-physiological and biochemical traits of quinoa, as well as soil fertility, under two water regimes (well-watered and drought stress) in open field conditions. Exposure to drought decreased biomass, leaf water potential, and stomatal conductance, and increased malondialdehyde and hydrogen peroxide content. Mycorrhiza and/or vermicompost promoted plant growth by activating photosynthesis machinery and nutrient assimilation, leading to increased total soluble sugars, proteins, and antioxidant enzyme activities in the leaf and root. After the experiment, the soil’s total organic matter, phosphorus, nitrogen, calcium, and soil glomalin content improved by the single or combined application of mycorrhiza and vermicompost. This knowledge suggests that the combination of mycorrhiza and vermicompost regulates the physiological and biochemical processes employed by quinoa in coping with drought and improves the understanding of soil–plant interaction.

## 1. Introduction

World agriculture currently is facing daunting and complex challenges along with the increase in the world population and rising incomes in developing countries. With dietary/lifestyle changes, this growth is driving up global food demand, which is expected to increase between 59% to 98% by 2050 [1,2,3]. At the same time, there is increasing pressures, from climate change, to urbanization, to a lack of investment, making it challenging to produce enough food [4]. The climate events—like droughts—change growing seasons, reduce water availability, limit crop productivity, and enable weeds, pests, and fungi to thrive in large ranges and numbers. These consequences will undoubtedly further affect food security, which must be ensured continuously in the coming years [3]. It is worth noting that drought stress in countries with dry climates, i.e., Australia, the Mediterranean region, the Midwestern US, and countries close to the equator, may substantially decline in agricultural output [5]. Altogether, doubling food production by 2050 will undeniably be a significant challenge.

First and foremost, farmers must adapt to climate change, and to meet the changes we all need to increase crop production and the expectations of regulators, consumers, food processors, and retailers. At the same time, farmers are under pressure to conserve water, mitigate the greenhouse gas emissions contributed to by agriculture, and to use fewer agricultural inputs. In this context, farmers worldwide will need to increase crop production either by (1) increasing the amount of agricultural land to grow crops, yet several countries, i.e., the Near East/North Africa and South Asia that have reached or are about to reach the limits of land available, or (2) by enhancing productivity on existing agricultural lands through (bio)-fertilizer and irrigation and adopting new methods/crops.

There is a strong scientific consensus that climate change-driven extreme weather, water scarcity, and increasing global temperatures will have severe long-term effects on crop performance and yields [6,7]. Indeed, water deficits have a devastating impact on major crop yield quantity and quality [8]. When plants are exposed to water scarcity, they undergo highly complex morphological, physiological, biochemical, and molecular changes [9,10,11,12]. In particular, drought can reduce the leaf area, water use efficiency, photosynthesis, and gas exchange [13,14]. It modifies the concentrations of plant proteins by reducing their content and suppressing their synthesis [15,16]. Reactive oxygen species (ROS) accumulate in plants during drought, and the plant tries to detoxify them by generating, among others, volatile derivatives, osmotic regulation, and/or the synthesis of a plethora of antioxidants [8,10]. In addition, physiological modelling studies predict that plant functional traits, such as the plant’s maximum photosynthetic capacity and the plant water transport/hydraulic tissues’ response to increasing water stress, will mediate the magnitude of water fluxes and how water fluxes change in response to soil [17]. Thus, belowground plant soil or microbiome traits could influence land–atmosphere interactions and could lead to more substantial drought intensification feedback [18,19,20]. Previous studies reported that the soil physicochemical properties change under water stress and may influence microbial communities’ composition [21,22,23]. In addition, dry soils contain low ions, including phosphorus (P), nitrogen (N), potassium (K^+^), calcium (Ca^2+^), and magnesium (Mg^2+^) [23,24].

As a relatively drought-tolerant crop for dryland agriculture in many countries, several farmers are switching to quinoa culture due to its wide geographic range [25,26,27]. Quinoa is recognized worldwide as an important gluten-free crop with high nutritional content and is a source of phytonutrients and fiber for human health. At present, little is known about the best management practices or the distinctive mechanisms evolved to cope with different droughts for quinoa on dryland farms. Agricultural tools for soil fertility to support growth are paramount to guarantee efficiency in terms of output over space.

Microbial-based and organic-based biofertilizers have emerged to improve crop performance by the exposure to specific conditioning and regimes [28,29,30]. Biofertilizers stimulate germination, root elongation, photosynthesis, the availability and absorption of soil nutrients, and soil microbial activity under stressful conditions [31,32]. Microbial biofertilizers, i.e., AMF, increase the availability of nutrients and phytohormones during their interaction with plant roots, while mitigating synthetic fertilizers and pesticides [33,34]. Under drought conditions, AMF extra-radical mycelia proliferate in the bulk soil beyond the rhizosphere and increase water and nutrient uptake, particularly P, which moves 10x faster in the mycelium than in the roots [35]. This symbiosis allows host plants to improve their performance by increasing stomatal conductance, photosynthesis, and osmolyte synthesis to maintain turgor pressure and the cellular functions necessary for metabolic processes under water stress situations [36]. The organic amendment application (i.e., compost, vermicompost, and biochar) has been demonstrated to improve crop resilience and yield [28,37,38]. The use of organic amendments could enhance C and the total organic matter content in the soil, as well as increasing mineral nutrition and the soil water-holding capacity [39,40,41]. Furthermore, applying organic fertilizers could enhance crop tolerance to environmental stresses by speeding up the process mediated by the microbe activity in the soil and enhancing the fungal-to-bacterial ratio in soil [42].

This study developed a successful field application of a biofertilizer-based approach to engineer plant-associated microbiomes and soil structures that can promote plant growth and health, as well as mitigating abiotic stressors, such as drought. In particular, a sustainable strategy, based on the effective endogenous AMF strain inoculation, composed of 15 species belonging to the Acaulosporaceae, Claroideoglomeraceae, Glomaceae, and Pacisporaceae families, as well as locally sourced vermicompost produced from horse manure mixed with straw that increases the ability to transform nutritionally essential elements from non-usable, to highly assimilable, forms without deleterious effects on the natural environment, was designed. Agro-physiological and biochemical responses of field-grown quinoa were used to gain mechanistic insight into the apparent effect of the vermicompost addition to a field soil, supplemented with AMF, upon its growth and tolerance. In the context of the drought-tolerance response, a potential role for ROS scavenging, water relations, and biochemical stress markers were highlighted, and that the rhizosphere and soil microbiomes represent an unparalleled resource for mitigating drought impact in quinoa development in agronomically consistent settings.

## 2. Results

### 2.1. Mycorrhizal Colonization and Plant Growth Improved in Field-Droughted Quinoa Treated with Biofertilizers

**Figure 1:**
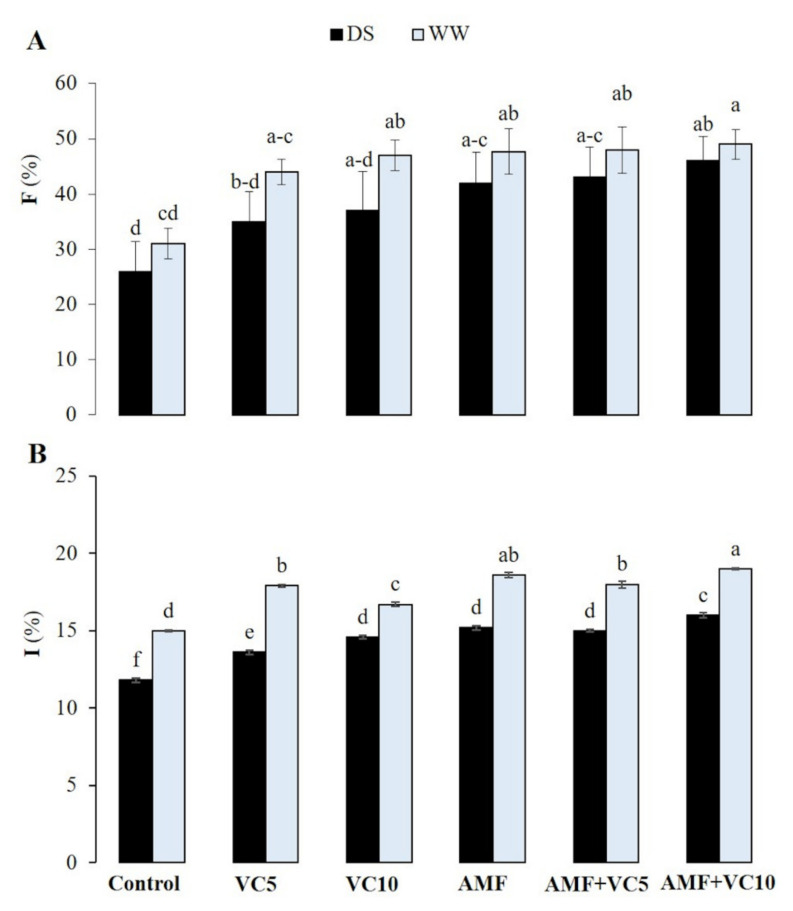
The effect of two water regimes (WW: well-watered and DS: drought stress) on (**A**) mycorrhization frequency and (**B**) mycorrhizal intensity in control plants (vermicompost-free and non-inoculated) and plants treated with vermicomposts (VC5: 5 tons/ha and VC10: 10 tons/ha) and inoculated with native arbuscular mycorrhizal fungi (AMF) alone or in combination. Means (±standard error) within the same graph, followed by different letters, are significantly different at *p* < 0.05.

Results showed that the frequency and intensity of the AMF infection in quinoa plant roots were significantly decreased by drought stress (*p* < 0.05). The un-inoculated and un-amended plants showed a mycorrhizal frequency in their roots of 31% under well-watered (WW), and 26% under drought stress (DS), conditions. The plants amended with vermicompost, VC10, alone or combined with AMF (AMF+VC10), showed the highest mycorrhizal frequency of approximately 50% under WW and 37% and 46%, respectively, under DS (Figure 1A). The mycorrhizal intensity was 15% in WW vs. 12% in DS in the control plants and 19% in WW vs. 16% in DS with AMF+VC10 treatment (Figure 1B).

**Figure 2:**
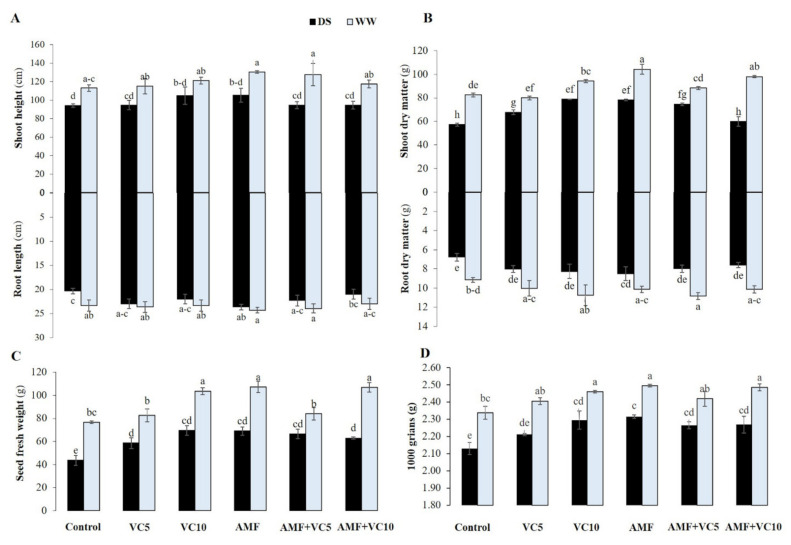
(**A**) Shoot height (upper panel) and root length (lower panel), (**B**) shoot (upper panel) and root (lower panel) dry matters, (**C**) seed fresh weight, and (**D**) thousand-grain weight of quinoa plants grown under two water regimes (WW: well-watered and DS: drought stress) after application (or not; control non-amended and non-inoculated) of vermicomposts (VC5: 5 tons/ha and VC10: 10 tons/ha) and native arbuscular mycorrhizal fungi (AMF) alone or in combination. Means (±standard error) within the same graph, followed by different letters, are significantly different at *p* < 0.05.

Drought stress showed a significant negative effect (*p* < 0.05) on plant growth by reducing plant height, root length, and the dry matter accumulation of shoots and roots (Figure 2A–D). The impact of drought stress was more pronounced in the shoot than in the root of quinoa plants. Drought stress showed a very significant reduction (30%) in control shoot dry matter (SDM) compared to VC10 and AMF+VC10 treated plants (approximately 5%), as well as WW plants (Figure 2B). Similarly, under DS, RDM decreased significantly by 26% in control plants, compared to WW plants (Figure 2B). AMF and/or VC (VC5 or VC10) applications significantly improved plant growth traits under DS conditions compared with untreated plants. The dual application of AMF and VC5 (AMF+VC5) performed better and better mitigated the effects of a water deficit on growth parameters compared to AMF+VC10. 

Under WW conditions, the single or dual application of VC10 and AMF recorded the highest values (approximately + 40%) of quinoa seed FW compared to the control plants (Figure 2C). Drought caused a significant decrease in this parameter, where the un-inoculated and un-amended controls showed a feeble response compared to the treated plants. 

### 2.2. Biofertilizers Mitigated the Adverse Effects of Field-Drought on Leaf Water Potential and the Efficiency of the Photosynthetic Machinery

**Figure 3:**
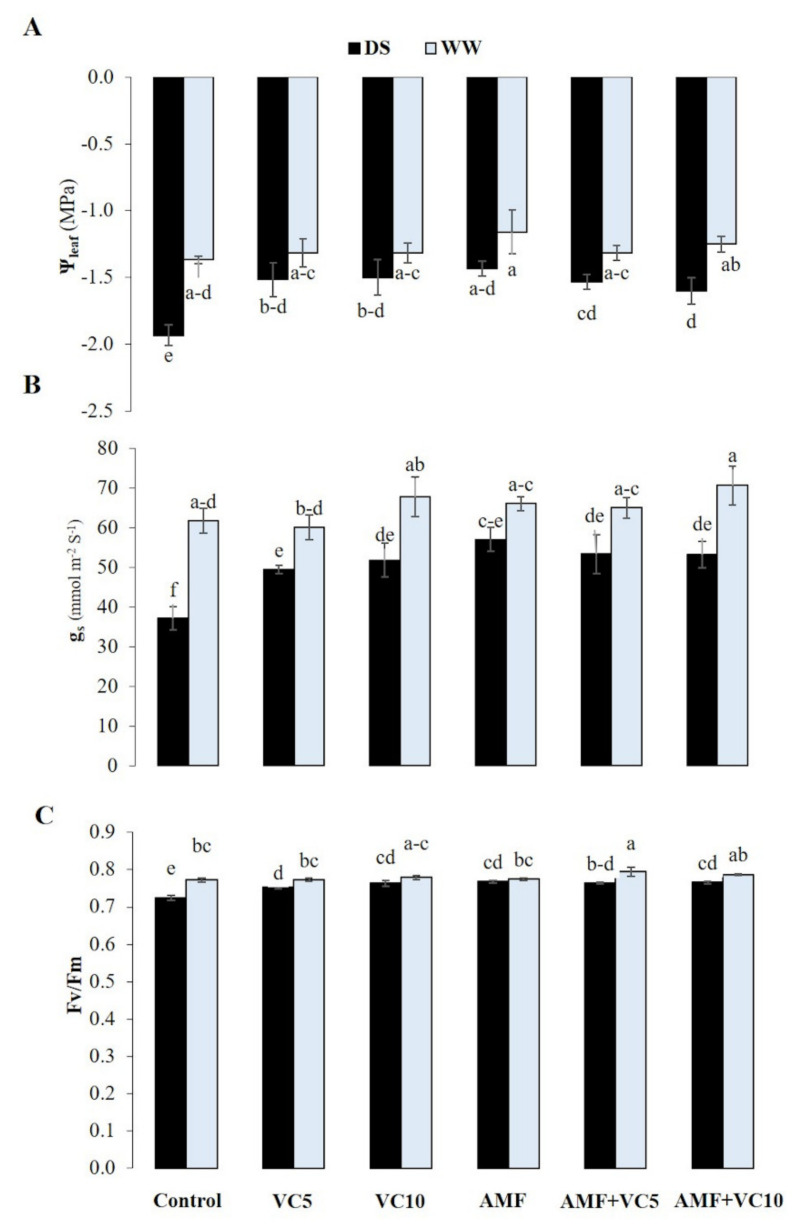
(**A**) Ψ_leaf_: Leaf water potential, (**B**) g_s_: stomatal conductance, and (**C**) F_v_/F_m_: chlorophyll fluorescence of quinoa plants grown under two water regimes (WW: well-watered and DS: drought stress) after application (or not; control non-amended and non-inoculated) of vermicomposts (VC5: 5 tons/ha and VC10: 10 tons/ha) and native arbuscular mycorrhizal fungi (AMF) alone or in combination. Means (±standard error) within the same graph, followed by different letters, are significantly different at *p* < 0.05.

Plants under field-droughted conditions suffered significant reductions in the physiological parameters (*p* < 0.05) (Figure 3), and this reduction was more pronounced in the control plants. AMF and/or vermicompost (VC5 and VC10) applications significantly enhanced Ψ_Leaf_, g_s_, and F_v_/F_m_ by 26%, 53%, and 6%, respectively, in AMF, and approximately 17%, 43%, and 6% in AMF+VC, respectively, compared to the water deficit untreated and un-inoculated treatment. 

**Figure 4:**
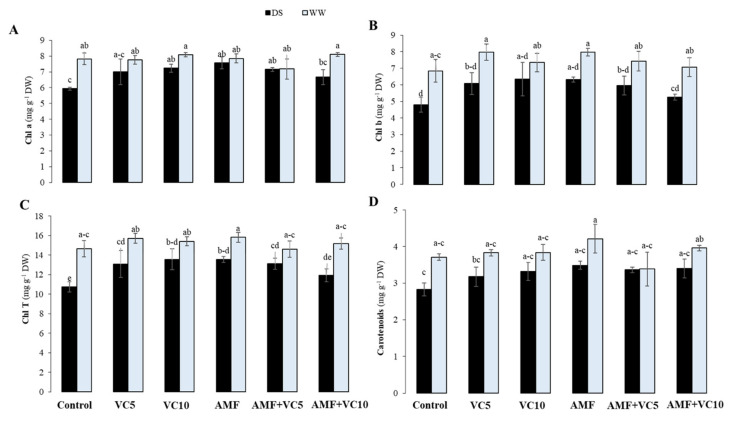
(**A**) Chl *a*: Chlorophyll *a*, (**B**) Chl *b*: chlorophyll *b*, (**C**) Chl *T*: total chlorophyll, and (**D**) carotenoid content in leaves of quinoa plants grown under two water regimes (WW: well-watered and DS: drought stress) after application (or not; control non-amended and non-inoculated) of vermicomposts (VC5: 5 tons/ha and VC10: 10 tons/ha) and native arbuscular mycorrhizal fungi (AMF) alone or in combination. Means (±standard error) within the same graph, followed by different letters, are significantly different at *p* < 0.05.

The water deficit promoted significant reductions in Chl *a*, *b*, total chlorophyll (Chl *T*), and carotenoids (*p* < 0.05) (Figure 4). However, the application of AMF and VC10 alone caused a significant increase in those pigments (28% and 22%, respectively, for Chl *a*; 31% and 32%, respectively, for Chl *b*; 26% and 26%, respectively, for Chl *T*; and 23% and 18%, respectively, for carotenoids) compared with the untreated drought-affected plants.

### 2.3. Biofertilizers Increased Total Soluble Sugar and Protein Content in Quinoa Plants

The total soluble sugar (TSS) and protein content of quinoa leaves and roots were significantly reduced under water deficit (Table 1). In contrast, the application of biofertilizers increased those parameters under both DS and WW in leaves and roots. Under DS conditions, the TSS and protein content increased by 85% and 50%, respectively, for AMF+VC10 in roots, and 21% and 26%, respectively, for VC10 in leaves compared to control plants.

### 2.4. The Oxidative Stress was Attenuated in Biofertilizer-Treated Quinoa Exposed to Water deficit

The H_2_O_2_ and MDA content in quinoa leaves and roots were increased by drought stress (Table 1). The control plants exposed to a drought deficit yielded the highest values of H_2_O_2_ and MDA: 30% and 35%, respectively, in leaves, and 23% and 43%, respectively, in roots. In contrast, the lowest H_2_O_2_ values were observed in plants treated with VC10 and AMF+VC10 by approximately 25% in leaves, and approximately 60% in roots, compared to the DS control plants. The combination of AMF+VC10 decreased the MDA content by 25% and 65% in leaves and roots, respectively (Table 1).

### 2.5. Antioxidant Metabolisms Improved in Drought Field Quinoa Treated with Biofertilizers

We examined changes in the activity of the antioxidant enzymes, including SOD, APX, POX, and PPO, in response to AMF and/or VC in quinoa plants grown under field drought stress. The antioxidant enzyme activities were significantly (*p* < 0.05) increased in DS compared to WW in quinoa plants (Table 2). The results indicate that under WW conditions, SOD, APX, and POX activities did not change in the leaf with biofertilizer application, while PPO showed a higher activity in leaves and a lower activity in roots with the microbial and/or organic fertilizers. DS conditions led to a significant increase in the enzymatic antioxidants. While SOD and APX increased in all treated plants, PPO and POX showed a lower leaf activity compared to controls in all the biofertilizer treatments. The AMF and/or VC application triggered a burst of POX and PPO activities in roots (Table 2). 

### 2.6. The AMF and Compost Applications Improves the Physicochemical Properties of Post-Harvest Agricultural Soil

For post-testing, the quality of the used agricultural soil, the pH value, the electrical conductivity (EC), the organic matter, the soil aggregate stabilizer (glomalin), and the nutrient contents were assessed. The data presented in Table 3 show that the organic matter, N, P, Ca, and glomalin production in the amended soil significantly increased at all amendment rates under WW conditions. In contrast, the Fe values decreased in all the treated plants, independently of the water regime. Under DS conditions, the pH in the postharvest soil improved with AMF inoculation, and EC was increased by approximately 25% and 20% in VC10 and AMF+VC10, respectively, compared to the untreated and un-inoculated controls. In addition, the biofertilizer application significantly increased TOM, P, and glomalin content compared to the control. In fact, under DS conditions, the highest values of TOM (33% and 32%) were yielded in soils treated with AMF+VC10 and VC5, respectively, and the highest value of P was recorded in soils treated with VC10 (1185%) and AMF+VC10 (1118%). Under DS conditions, VC and AMF improved both soil T-GRSP and EE-GRSP contents, compared to the control. The highest T-GRSP increments were observed in soils treated by VC5 (148%) and AMF+VC5 (130%). The Ca concentration showed a significant decrease in soils treated with VC10 and AMF+VC10 under DS conditions. Under the same conditions, VC10 (75%) and VC5 (42%) treatments improved the N concentration compared to the stressed control, while the Fe concentration recorded its highest value in the control treatment. 

### 2.7. Principal Component Analysis and Heatmap of Quinoa Traits in Response to Biofertilizers and Water Scarcity

**Figure 5:**
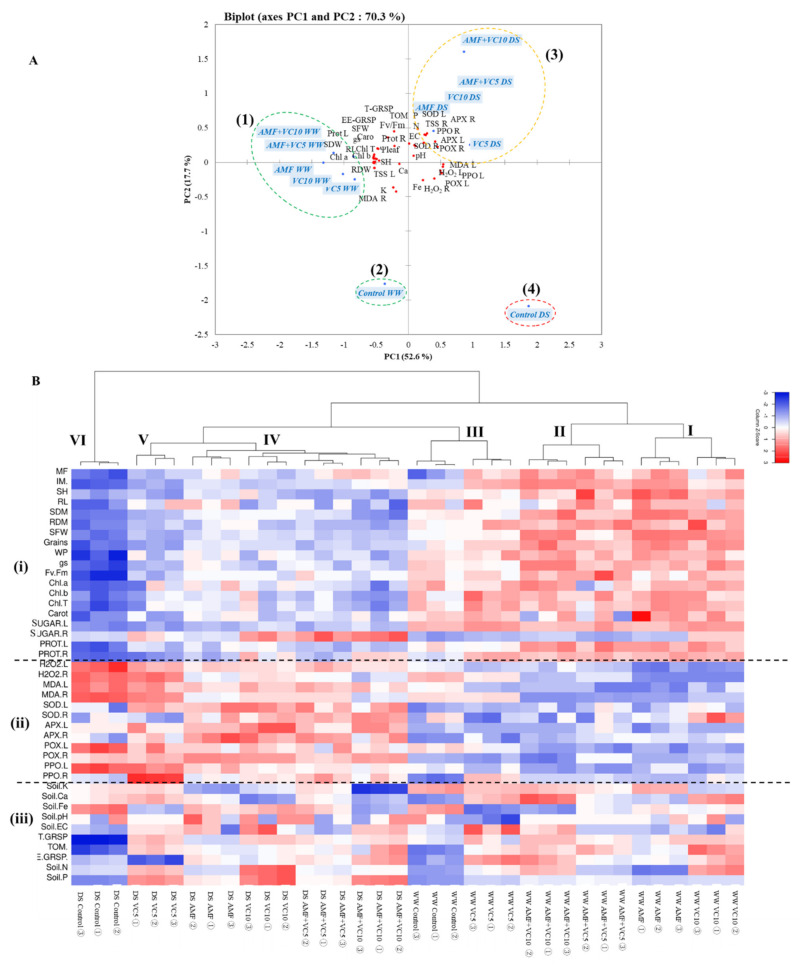
(**A**) Principal component and (**B**) Heatmap analyses of quinoa plants (leaf and root) subjected to different treatments. The variables (agro-physiological, biochemical, and soil traits) are represented in red. The treatments are given in blue. Ψ_Leaf_: leaf water potential; AMF: arbuscular mycorrhizal fungi; APX L: ascorbate peroxidase in leaf; APX R: ascorbate peroxidase in root; Ca: calcium; Car: carotenoids; Chl *a*: chlorophyll *a*; Chl *b*: chlorophyll *b*; Chl *T*: total chlorophyll; EE-GRSP, easily extractable glomalin-related soil protein; F: frequency of mycorhization; Fe: iron; F_v_/F_m_: chlorophyll fluorescence; g_s_: stomatal conductance; H_2_O_2_ L: hydrogen peroxide in leaf; H_2_O_2_ R: hydrogen peroxide in root; I: intensity of mycorhization; K: potassium; MDA L: malondialdehyde in leaf; MDA R: malondialdehyde in root; N: nitrogen; P: phosphorus in soil; POX L: peroxidase activity in leaf; POX R: peroxidase activity in root, PPO L: polyphenoloxidase activity in leaf; PPO R: polyphenoloxidase activity in root; Prot L: protein in leaf; Prot R: protein in root, RDM: root dry matter; RL: root length; RWC: relative water content; SDW: shoot dry weight; SH: shoot height; SOD L: superoxide dismutase activity in leaf; SOD R: superoxide dismutase activity in root; T-GRSP, total glomalin-related soil; TSS L: total soluble sugar in leaf; TSS R: total soluble sugar in root; VC: vermicompost.

To evaluate the contributions of each parameter in the control and biofertilizer-treated quinoa plants, we performed a PCA using morphological, physiological, biochemical, and post-harvest soil parameters collected from plants after WW and DS treatments. The PCA showed that those treatments (in blue) and the variables (in red) were associated with two top PCs, accounting for 70.3% of the total variation of the traits under the field conditions (Figure 5A). PC1 explained 52.6% of the total variation and was strongly influenced by the antioxidant enzymes, while PC2 accounted for 17.7% and was strongly associated with the growth, soil-related, and photosynthetic machinery parameters (Figure 5A and Supplementary Table S1). Under WW conditions, the PCA showed a positive correlation among the treatments applied separately or in combination on soil fertility, growth, photosynthetic pigments, and sugar and protein content in leaves and roots, which were positively correlated with each other. The analysis also confirmed the negative impact of DS conditions on these parameters. The biplot revealed a negative correlation between the control DS treatment with H_2_O_2_ content, and the leaf antioxidant enzyme activity. Under DS conditions, the PCA showed a clear positive correlation between the enzymatic antioxidants, pH, and EC with AMF applied alone or combined with vermicompost (VC10 or VC5).

Similarly, the PCA showed that all biofertilizer treatments, single or combined under WW or DS, were separated from their controls (Figure 5A). The growth, physiological, biochemical, and soil parameters grouped the applied treatments into four major groups. the best treatments, in terms of more growth and an efficient photosynthetic apparatus, are on the left side of the first axis (PC1), corresponding to the treated (1, upper left panel) and untreated control (2, lower left panel), were grown under WW conditions. In contrast, the drought-stressed quinoa (right side of the first axis) was separated into two sub-groups, which were represented by biofertilizer-treated plants (3, upper right panel) which corresponded with intermediate growth, a better tolerance to DS, and an effective enzymatic system. The control treatment without inoculation (control DS) (4, lower right panel) showed lower growth and a higher accumulation of the oxidative stress markers, MDA and H_2_O_2_.

To identify the key parameters for assessing the impact of AMF and/or VC as drought tolerance inducers in quinoa, agro-physiological and morphological measurements were used to plot a heatmap. As shown in Figure 5B, an HCA derived from a two-way hierarchical clustering analysis showed that all the growth, physiological, biochemical, and post-harvest soil physicochemical traits of treated and untreated quinoa grown under WW and DS could be clearly separated into six major clusters (Figure 5B). When grown under WW conditions, the treatments clustered into three subgroups (I, II, and III) while the same set of treatments grown under drought conditions clustered into separate sub-groups (IV, V, and VI) (Figure 5B, upper side). This apparent clustering demonstrates that in comparison to control conditions, and independently of the water regime, biofertilizer treatments alter the morphological, physiological, and biochemical characteristics of quinoa. Interestingly, it should be noted that the analysis of different parameters (Figure 5B, left side) showed three main groups where group (i) represents key morphological, physiological, and growth parameters; group (ii) includes the antioxidant enzyme activity, as well as the contents of MDA and H_2_O_2_ associated with drought susceptibility/tolerance; and group (iii), which represents the soil minerals and glomalin-related soil proteins. The values of SH, RL, SDW, RDW, leaf water potential, F_v_/F_m_, pigments, TSS, and proteins, as well as minerals (P and N), were moderate under water stress conditions in the plants treated with AMF and/or vermicompost, compared to the control plants under WW conditions. In contrast, MDA and H_2_O_2_ showed a contrasting effect under DS conditions in the control plants’ leaves and roots.

## 3. Discussion

Drought alone causes more global yield loss than all pathogens combined [43]. The strategy highlighted here can help to offset the economic losses caused by drought by increasing the crops’ tolerance. However, to facilitate the widespread adoption of these technologies in agriculture, their potential and reliability need to be validated in the field to ensure that they remain affordable and more effective than the existing approaches. Previous studies demonstrated the role of AMF on drought and salt stress mitigation in alfalfa, date palm, and carob plants [44,45,46]. However, the role of native AMF in combination with vermicompost on alterations in ROS scavenging machinery, to impart tolerance against field drought stress, has not yet been studied. In this light, the present investigation delves into the impact of AMF and horse manure-based vermicompost alone, or in combination, on soil parameters, growth, biomass, gas exchange, water relations, metabolite accumulation, and oxidative damage in quinoa in field trials.

Drought stress caused a significant decrease in the growth of quinoa, particularly when non-inoculated with AMF and compost. Several studies showed that water stress causes a reduction in growth, biomass, and the yield of pseudo-cereals [47,48]. Drought stress can reduce the leaf area, chlorophyll content, photosynthesis performance, stem diameter, plant height, grain yield, and stomata opening [49,50,51], as well as reducing the nutrient uptake by the roots and their transport to the stem [52]. Sun et al. [53] reported that the water limitation significantly reduced quinoa growth, biomass, and seed yield by 26% compared to well-watered ones. Our results revealed that AMF alone or in combination with VC significantly improved growth parameters, including plant height, root elongation, shoot and root biomasses, and the seed yield of quinoa in both WW and DS conditions. Under WW conditions, the growth trait improvement in AMF and VC treatments were in accordance with previous studies [54,55]. Hussin et al. [56] revealed that AMF significantly increased quinoa shoot and root DM compared to control plants under DS conditions. Under DS conditions, the growth and yield of quinoa were significantly improved by applying VC at the rate of 10 t/ha. Previous studies reported that the organic amendment significantly enhanced the growth and yield of crops grown under water limitation, including wheat [57,58], quinoa [59,60], rice [61], tomato [62], date palm [46], and blackgram [63]. Boutasknit et al. [64] reported that the dual biofertilizers, AMF+VC, enhanced plants’ above- and below-ground systems. The observed increase in performance and yield traits in VC-treated quinoa can be linked to the mineral nutrition and solubilization of mineral nutrients [65,66]. Likely, the beneficial effects of the inoculation by the native AMF are attributed to nutrient recycling, mineral nutrition, the solubilization of nutrients (i.e., P, K, and Fe), the biodegradation of soil organic matter, phytohormone production, antibiotics production, soil structure, aggregation improvements, and plant resistance to pests and diseases. Under DS, AMF can help promote aquaporin synthesis, which can enhance water uptake and water homeostasis maintenance [67,68]. AMF, exclusively found in the native habitat of wild relatives of crop plants, is comprised of the representatives of the *Glomus* species, known for its maximum germination, greater absorption of minerals and water under soil drying conditions, and its capabilities to produce bioactive compounds. *Acaulospora* members, generally known to produce a lot of mycelia, as well as *Claroideoglomus* and *Rhizophagus,* are recognized for the high root infection intensity, nutrient cycling, and efficiency in providing P to roots.

Furthermore, several species of microorganisms in the compost increases nutrient mineralization, which, in turn, improves nutrient uptake by plants [69]. The humus, C, P, and N existing in the compost improves soil fertility by increasing the essential mineral nutrients for plant growth and development [58]. Perner et al. [70] reported that AMF symbiosis could increase the uptake of P and N, showing a direct benefit on plant growth when organic fertilizers have been used. Nutrient availability, including P in the plant rhizosphere, can influence photosynthesis, protein biosynthesis, membrane transport, and the division and elongation of cells, resulting in plant biomass accumulation [71,72]. Data has shown that the addition of compost at 10 t/ha could be considered as the optimal dose for quinoa growth in the presence of AMF under drought conditions. Providing 10 t/ha, and a predictable supply of “slow-release” nutrients, allows the AMF colonization of roots and their establishment, which could stimulate the functioning of the compost–AMF complex, especially in low nutrient soils [39]. The enrichment of poor inorganic matter soils by compost-derived substances can release and store nutrients and promote plant growth and biological activities, as well as provide a source of energy for microorganisms, which can modulate the structure, physicochemical properties, and aeration of the soil [40,73,74]. In this context, our results revealed that the dual combination of microbial inoculation and soil amendment with compost improved the glomalin-related soil proteins. AMF released glomalin, which also contains polysaccharides, a sticky substance that forms hydrophobic interactions with the soil particles to glue aggregates together, as well as iron to form stable bridges with clay minerals [75]. Miller and Jastrow [76] showed that GRSP later caused the aggregation of clay, silt, and other particles, as well as bringing together nutrients and biota. This ‘extra’ uptake of nutrients promotes root and shoot development, plant height, and biomass accumulation under DS conditions [77,78,79]. It should be noted that the AMF+VC10 application generally had a better performance in plant growth and AMF colonization under drought stress. The unique AMF extraradical mycelia act together, like a net, firmly holding the soil enriched structure, increasing the soil water holding capacity, and limiting leaching, indirectly improving soil aeration [80]. This enriched soil structure and improved soil health (Table 1 and Table 2) around the root area, induced by AM fungi and compost, ultimately translates to healthier quinoa crop growth, and helps to achieve a maximum yield.

As in our study, drought can negatively affect mycorrhizal colonization in quinoa [56,81]. Similarly, previous studies revealed that root colonization by AMF and hyphal growth declined under water limitation stress [44,82,83]. The soil water status significantly affects germination and/or spore development [84,85]. This finding could be, at least partially, explained by the fact that plants reduce their C allocation to soil and roots under the water limitation stress, which would restrict the formation of AM symbiosis and the extension of AM fungal mycelia [86,87,88,89]. Notwithstanding, under DS conditions, VC additions, particularly with a rate of 10 t/ha, enhanced root colonization compared with plants treated with AMF alone under DS conditions, an observation supported by previous studies [54,79,90,91,92]. These results can be explained by the stimulating effect of VC on the biological activity of AMF [55]. Furthermore, VC contains a large amount of humic acids that may stimulate the hyphal activity of AMF [93,94].

A water deficit negatively affects physiological traits and limits quinoa growth. Plants have strategies, such as improving their antioxidant system (Table 2), to prevent water loss, balance the optimal water supply to vital organs, maintain cellular water content, and persevere through periods of drought. However, the increments of enzyme activities in control plants under DS seems insufficient to counterbalance the H_2_O_2_ accumulation and prevent tissue damage (Table 1), hindering its survival. The application of biofertilizers, mainly AMF+VC, endures drought tolerance with increased internal water content (Figure 3), preventing tissue injuries (Table 2) while sustaining growth (Figure 2) over the drought period. The improvement in morphological and physiological characteristics under DS conditions is consistent with the improvement in the root colonization of quinoa plants through the addition of VC. Under water limitations, mycorrhizal hyphae mediate the absorption and water transport from the soil to the host plant by regulating the stomatal activities, photosynthetic machinery, and antioxidant activities [95,96]. At the cellular level, AMF and/or compost mediate the drought signals that promote stress-protectant metabolite production, triggering the antioxidant system to maintain redox homeostasis, and can deploy peroxidase enzymes to reduce acute cellular damage and membrane integrity. Non-amended/non-inoculated plants grown under DS showed decreased g_s_, which could be explained as a more rapid defense against dehydration. The application of biofertilizers increased water uptake, g_s_, and F_v_/F_m_, and reduced the degradation of chlorophyll and carotenoids under drought stress. This improvement in physiological traits suggests a better photosynthetic apparatus performance, leading to increased CO_2_ assimilation for photosynthesis. Compared to other composts, the VC contains higher macro-and micro-nutrients [97,98]. Applying VC (10 t/ha) alone or in combination with AMF improves the absorption of water and nutrients, i.e., N, Mg, K, Ca, and P, which are involved directly or indirectly in the opening of stomata and the functioning of various photosynthesis compounds under drought stress [99]. Our previous studies revealed that the increase in physiological traits (Ψ, g_s_, F_v_/F_m_, and chlorophyll pigments) in amended and/or inoculated plants is due to the hydraulic conductance increase, the root absorption surface area, and the osmotic adjustment [29,44].

During drought, biofertilizer-treated plants showed lower potentials and higher water content levels, allowing the inoculated and amended plants to sustain high organ hydration and turgor level, which maintained cells physiological activities, especially those linked to the photosynthetic apparatus. Recently, Quiroga et al. [100] revealed that AMF hyphae could replace the role of aquaporin activity under DS conditions. The root aquaporins showed no dramatic changes or down-regulations under the water shortage of inoculated plants [82]. This showed that the AMF-colonized roots actively maintained the physiological water balance during drought spells [101]. Roots respond to changes in soil moisture at the cellular level and with the entire root system architecture. The root system architecture undergoes morphological changes during water limitation to improve its ability to absorb water and nutrients [102,103]. It has been shown that AMF exudates, which are involved in the dialogue between AMF and their host during the pre-colonization phase, play roles in the cell division, elongation, and differentiation events in the root apex and in lateral root formation [104,105]. In the pursuit of moisture, root systems grow differentially in the presence/absence of biofertilizers and adapt their architecture to be either deep or shallow (Figure 2). Longer and deeper roots, as observed in biofertilizer-treated plants, can efficiently capture soil water, and retain moisture in deep layers [43]. In the same way, the application of organic amendments induces similar changes by enhancing the soil water holding capacity and nutrient content [106].

Drought can cause the formation and accumulation of ROS, which are usually generated through a number of metabolic pathways [107]. ROS affect many cellular functions by causing damage to nucleic acids, the oxidation of proteins, and lipid peroxidation, which is considered the most damaging process known to occur in every living organism [108]. The enhancement of the H_2_O_2_ generation in plants is often observed in response to stresses [44]. In drought conditions, roots sense the water shortage from the soil. The aboveground segments of plants respond by stomatal closure, implicating a systemic communication system. Our results showed that the soluble sugar and soluble protein contents of treated plants subjected to drought stress were greater than those of untreated plants, or under WW conditions. The biofertilizer-treated plants’ response to water deficit stress is linked to the increase in solutes’ net concentrations, or osmotic adjustments, and is an essential mechanism for maintaining cell water content and, thus, turgor. An increase in the total soluble sugar contents with drought could be due to starch accumulation and the total soluble sugar in quinoa under stressed conditions. These results agree with other findings that reported that wheat inoculated with AMF increased the carbohydrate and protein content compared to untreated plants [109]. Under water stress, the accumulation of the total soluble sugar could protect plant cells from osmotic damage [110,111,112,113]. The sugar content in inoculated and amended plants was higher than those in non-inoculated and non-amended plants, thereby confirming that the biofertilizers’ application activates the natural physiological metabolisms under DS [46]. Ahanger et al. [114] and Bárzana et al. [49] revealed an upregulation of the sugar metabolism-related genes in AMF-treated plants under stress conditions. Singh et al. [115] found that the inoculation with an AMF consortium, containing nine AMF species, increased protein levels in tea tree leaves. Under DS, quinoa plants inoculated with AMF accumulated higher proteins and sugar content, likely to maintain high hydration and turgor levels and to maintain the overall physiological traits [95,116]. In addition, the higher content of soluble proteins in inoculated and amended plants may explain the strengthening of the enzymatic antioxidant defense system under abiotic stress [28,45,46].

Membrane damage is taken as a single parameter to determine the level of lipid destruction under stress conditions. Lipid peroxidation occurs when the above-threshold ROS levels are reached, directly affecting normal cellular functioning and aggravating oxidative stress by producing lipid-derived radicals and decreasing membrane stability [117,118]. Under DS, the increased ROS levels likely resulted from chloroplast damage and mitochondrial electron transport chain disruptions (Figure 3 and Figure 4), leading to the breakdown of proteins (Table 1) by oxidative reactions or by proteolytic activities. Additionally, DS increased the MDA and H_2_O_2_ content (Table 1 and Figure 5) in quinoa leaves and roots, regardless of the treatment applied. This result was consistent with previous findings reporting an H_2_O_2_ content increase in wheat with lowering soil humidity [119]. A water deficit increases the production and concentration of H_2_O_2_, which causes protein denaturation, lipid oxidation, and DNA damage [120]. This damage affects the growth and development of quinoa leaves and roots, particularly in non-amended and non-inoculated plants. However, the application of VC alone, or combined with AMF, lowered MDA and H_2_O_2_ production compared to untreated quinoa subjected to drought, suggesting that AMF symbiosis and/or VC (at 10 t/ha) could help quinoa plants to reduce the oxidative damage in response to water insufficiency.

Our results then suggested that the positive effects of AMF colonization and VCs on the plant antioxidant system might have enhanced antioxidant enzyme activity in the quinoa organs. The antioxidant enzymes and specific metabolites play an important role in scavenging ROS concentrations and minimizing oxidative stress in plant cells. Further studies have indicated that various antioxidant enzymes are indispensable for the cellular defense strategy towards excessive ROS production in plant cells [121]. The changes in the activities of the antioxidant enzymes, including SOD, APX, POD, and PPO, in response to droughted quinoa in the presence/absence of biofertilizers, was examined. The increased activities of these antioxidant enzymes in untreated plants, in both the leaves and roots, under DS compared to WW conditions, did not provide enough protection against ROS as shown by the simultaneously excessive oxidative stress (H_2_O_2_ and MDA contents). Therefore, an enhanced antioxidative metabolism could increase the capacity of plants to scavenge ROS, only to a certain extent. Together with the lower H_2_O_2_ and MDA contents detected in mycorrhizal and VC-treated plants, relative to the non-mycorrhizal and VC-free plants, it is conceivable that biofertilizers protect plants against drought by enhancing SOD, APX, POX, and PPO activities in quinoa leaves and roots. Wang et al. [121] found a positive correlation between the high activity of antioxidant enzymes and water stress alleviation. These results align with research by Anli et al. [46] which indicated that stressed plants contain higher antioxidant enzymes concentrations than non-stressed plants. Indeed, our study showed that the activity of antioxidant enzymes was greater in the leaves than in the roots of quinoa. This may be because these enzymes are present in large quantities in the chloroplasts [122]. Our data revealed that the quinoa drought tolerance mechanisms were further improved in plants grown in the presence of VC at 10 t/ha, as well as AMF, which helped plants scavenge ROS and helped to maintain the antioxidant level in the quinoa. This ‘manoeuvre’ is likely achieved through the AsA-GSH cycle involving various antioxidative enzymes and electron donors. However, further studies are required to decipher the relation between the biofertilizer–metabolic and antioxidative system pathways in plants.

Among the several disposal strategies, as noted above, the application of biofertilizers have a myriad of benefits to agricultural soils. In addition to improving the growth of quinoa plants, the current research aimed to study, at postharvest, the value of the soil application of AMF and/or VCs in amending soil fertility without threats to the environment. Our analyses indicated that the preliminary analysis of the agricultural soil showed low TOM and micronutrients (Table 4), and the application of the proposed biofertilizers improved the soil physicochemical properties and, most importantly, the recycling of valuable nutrients for plants (Table 4). After four months of quinoa cultivation under WW and DS conditions, an improvement in pH, EC, TOM, P, and glomalin content was recorded after biofertilizer application, suggesting the use of VCs and AMF as a replacement for chemical fertilizers. These results are probably due to the richness of vermicompost in organic matter (61%) and the role of AMF in metabolizing different compounds produced by plant roots [123]. The soil richness in the organic matter could ensure a good supply of major elements (i.e., N, P, K, Ca, and Fe) through the mineralization process [123]. Furthermore, AMF could improve the chemical and nutritional quality of the soil through different mechanisms, including P solubilization, soil structure, and aggregates, via the glomalin liberation. Seminal works showed the direct relationship between soil glomalin content and soil physicochemical properties [45,93,123,124]. Intriguingly, K, Ca, and Fe quantities in the postharvest field soils were lower in the biofertilizer-treated treatments. These data could be, at least partly, explained by their absorption into the mycorrhizal and VCs-treated quinoa, which recorded higher growth than the control. It is worth mentioning that the use of biofertilizers (rich in nutrients and bioactive compounds) have also been used in hydroponics (termed as ‘bioponics’) for healthy plant nutrition. In this context, its use in hydroponic crops can be an environmentally friendly alternative for the supply of nutrients, particularly for relatively short-cycle crops such as lettuce and can also significantly increase the fruit-to-vegetative growth ratio in crops, such as the tomato [125,126].

Altogether, the PCA and correlation analyses showed that the differences in drought tolerance among the treated quinoa (or not treated quinoa) were mainly due to variations in the agro-physiological and biochemical parameters. The results indicated that the physiological, morphological, and biochemical parameters contributed more than the soil-related traits, separating the control and drought-treated groups. This suggests that the agro-physiological characteristics may be closely associated with drought tolerance in quinoa (Figure 5). These data suggest that the biomasses (SDM and RDM), stress markers (H_2_O_2_ and MDA), and enzymatic antioxidant (SOD, APX, and PPO) parameters are reliable indicators for screening effective treatments for tolerance to water deficits during quinoa growth. Nevertheless, as no comprehensive/standardized system for evaluating drought resistance has been established, yield loss indices (despite their labor-intensive and time-consuming nature) under water scarcity conditions, compared to normal conditions, have been used in crop breeding programs [127].

## 4. Materials and Methods

### 4.1. Field Set-Up, Experimental Protocol, Plant Material, and Treatments

The field experiments were conducted nearby Marrakesh, Morocco (31°37′39.9′’ N and 08°07′46.7′’ W). The region’s climate is semi-arid, with an average annual rainfall of 250 mm (from September to June) and an average temperature of 27.6°C. The fields have never been treated before with chemical fertilizers or other organic fertilizers, and the physicochemical parameters of the agricultural soil (AS) are shown in Table 4.

Two watering conditions were subsequently used on plots: I) well-watered (WW), consisting of regular watering (approximately 8 L/h), with the first irrigation starting in Week 1 (February 8) and continuing until the sampling, and II) drought stress (DS), consisting of a lack of irrigation (50% water reduction; approximately 4 L/h). The field sites were irrigated at five-day intervals using drip irrigation system lines, with appropriate internal drippers, placed on the soil surface of each furrow. When irrigated, WW plots received 100% of the weekly calculated crop evapotranspiration for the five days before each irrigation. Crop evapotranspiration was determined according to Naylor et al. [128] using the potential evapotranspiration multiplied by the crop coefficient, which was adjusted according to the crop growth stage.

Each water regime (WW or DS) was comprised of six treatments: (1) Control: non-inoculated and non-amended; and (2) AMF: seedlings that were inoculated (2 g/plant, for a total of 160 g/plot) with an indigenous consortium of AMF (containing mycorrhizal roots, substrate, and AMF spores). The AMF consortium was collected and isolated from the rhizospheric soil of the Tafilalet palm grove, located 500 km southeast of Marrakesh (Morocco) ([96]; see below); (3) VC5: seedlings amended with vermicompost at 0.6 kg/plot (5 t/ha). The VC was produced in two different phases: the pre-composting part consisted of a mixture of 75% horse manure and 25% straw in a windrow for 45 days. After reducing the temperature to 25 °C, earthworms (*Eisenia fetida*) were added to the mixture with a density of 50 worms per liter (10 g worms/kg mixture). This second phase was carried out in a greenhouse for 50 days (a total of 95 days to run the vermicomposting). The physicochemical properties of vermicompost are presented in Table 4; (4) VC10: seedlings amended with the same vermicompost at 1.2 kg/plot (10 t/ha); (5) AMF+VC5: joint application of AMF and VC5; and 6) AMF+VC10: joint application of AMF and VC10.

The experimental conditions were assigned via a randomized block design, where the fields were divided into 36 plots (0.8 m wide separated from each other by 0.5 m) of 6 rows each (approximately 60 plants per plot). Each plot was randomly allocated a watering regime (WW or DS) comprising of biofertilizer treatments (or not), with three replicates, for a total of 36 (2 × 6 × 3) plots. At harvest time, ten representative plant samples from each plot were collected. Within each plot, individual plant samples of leaves and roots were manually collected at the same time of the day (10 am to 1 pm) at the flowering stage. The leaves, from 10 representative plants, were collected by tearing off the third and fourth fully emerged leaf from each plant, pooling material into an aluminum foil bag, and then flash-freezing them in liquid nitrogen. Roots were collected, from the same plant, using a shovel at 30 cm deep into each plant’s system, avoiding the brace roots, were cut using garden shears, were kept in an aluminum foil bag, and were then pooled. Roots were vortexed for 2 min in an epiphyte removal buffer [129] to remove rhizosphere soil, were rinsed twice in a root washing buffer, gently dried, placed in aluminum foil bags, and were quickly frozen in liquid nitrogen.

### 4.2. Mycorrhization Assessment

The extraction, counting, and identification of AMF consortium spores were based on dry soil (100 g) collected from the palm grove [96,130,131,132,133], International databases: https://invam.wvu.edu (accessed on 1 January 2022) and http://www.amf-phylogeny.com (accessed on 1 January 2022). Spore extraction was made as described by Gerdemann and Nicolson [130].

The mycorrhizal consortium was composed of 15 species: *Acaulospora delicata*, *Acaulospora leavis*, *Acaulospora* sp, *Claroideoglomus claroideum*, *Glomus aggregatum*, *G. claroides*, *G. clarum*, *G. deserticola*, *G. heterosporum*, *G. macrocarpum*, *G. microcarpum*, *Glomus* sp, *G. versiforme*, *Rhizophagus intraradices*, *Pacispora boliviana*. These species belong to four families (Acaulosporaceae, Claroideoglomeraceae, Glomaceae, and Pacisporaceae), which include five genera: Acaulospora, Claroideoglomus, Glomus, Rhizophagus, and Pacispora. The genus Glomus has the highest percentage (60%) of species, followed by Acaulospora (20%), and the genera Claroideoglomus, Rhizophagus, Pacispora, with a low percentage (6.7%). This AMF consortium was developed in Zea mays L. roots for three months. Corn roots containing hyphae, vesicles, and spores were harvested, cut into small pieces, and used as the inoculum. The number of AMF spores detected in this inoculum was 47 spores/100 g of the soil sample.

Quinoa roots were washed, cleaned with 10% KOH at 90 °C for 20 min, and were then placed in lactic acid for 10 min at room temperature. Subsequently, they were stained with 0.05% trypan blue at 90 °C for 20 min, according to Phillips and Hayman [134]. Root fragments of 1 cm long were observed in the glycerol droplet. The frequency (F%) and intensity (I%) of root colonization were calculated microscopically according to the method of Derkowska et al. [135], using 20 randomly selected root fragments, repeated five times for each sample.

F (%) = 100 × (infected root segments/total root segments)

I (%) = ((95 × n5) + (70 × n4) + (30 × n3) + (5 × n2) + n1)/total root segments,

Where n represents fragments with an index of 0, 1, 2, 3, 4, or 5, with the following infection rates: 100 > n5 > 90; 90 > n4 > 50; 50 > n3 > 10; 10 > n2 > 1; 1 > n1 > 0.

### 4.3. Plant Performance

Four months after planting, the shoot height, root length, shoot and root dry matter, and fresh seed weight were measured. All plant samples’ dry matters were determined after oven drying at 70 °C until the weight remained constant. Moreover, the thousand-grain weight in each plant was determined.

### 4.4. Physiological Investigations

#### 4.4.1. Leaf Water Potential

Leaf water potential (Ψ_Leaf_) was determined using a pressure chamber (SKPD 1400, Skye Instruments, Powys, UK) at predawn (6 am-8 am). The measurements were taken on fully developed leaves from the upper stem. The Ψ_Leaf_ were measured over the same day and immediately after gas exchange measurements.

#### 4.4.2. Stomatal Conductance, Chlorophyll Fluorescence, and Chlorophyll Pigment Measurements

Fully expanded leaves were used to measure chlorophyll fluorescence (F_v_/F_m_), and stomatal conductance (g_s_). F_v_/F_m_ was measured using a modulated chlorophyll fluorometer (model OSI 30p, Opti-sciences). The dark adaptation was made on the upper side of the third fully developed leaves, located at the middle third of the plant, and was adapted to obscuring for 30 min using leaf clips before measurements were taken. This parameter was measured by transmission at 650 nm on a leaf area of 12.5 mm^2^. The F_v_/F_m_ values represent the quantum yields (F_v_/F_m_ = (F_m_ − F_0_)/F_m_)), where Fm and F0 are the maximum and initial quantum yields of dark-adapted leaves, respectively [136]. Stomatal conductance was measured using a porometer (leaf porometer, model SC1) as described by Harley et al. [137]. F_v_/F_m_ and g_s_ measurements (20 per plot) were made on the abaxial part of each plant between 9:30 am and 11:00 am on a sunny day. The extraction and quantification of the photosynthetic pigments (chlorophyll *a*, chlorophyll *b*, and carotenoids) were done by grinding leaves (0.5 g) in 80% cold acetone, with centrifugation at 12,000× *g* for 20 min. The supernatant absorbance was read at 480, 645, and 663 nm [138].

### 4.5. Measurements of Total Soluble Sugar and Protein Contents

The total soluble sugar (TSS) content in the leaves and roots of quinoa was estimated according to the phenol sulphuric acid method [139]. Glucose was used as a standard. Briefly, samples (0.1 g) were ground in liquid nitrogen and then ethanol (80%) and centrifuged at 5000 rpm for 10 min. The supernatant (0.25 mL) was mixed with 0.25 mL of phenol and 1.25 mL of sulfuric acid. The TSS content was then determined by measuring the absorbance at 485 nm. TSS was determined as mg g^−1^ DM, using a calibration curve (40, 80, 120, 160, and 200 µg mL^−1^ of glucose).

The soluble protein contents were defined by using the method of Bradford [140]. Plant samples (1 g) were homogenized with 4 mL of 1 M phosphate buffer (pH 7.2) and were then centrifuged at 18,000× *g* for 15 min at 4 °C. Supernatants and dyes were pipetted in spectrophotometer cuvettes, and absorbance was read at 595 nm.

### 4.6. Quantification of Lipid Peroxidation and Hydrogen Peroxide

Stress indicator (hydrogen peroxide (H_2_O_2_) and lipid peroxidation as malondialdehyde (MDA)) equivalents were extracted from the leaf and root tissues. MDA was determined by estimating the TBA reactive substances (TBARS), as described by Madhava Rao and Sresty [141]. Samples (0.1 g) were homogenized in 3 mL of 0.1% (*w/v*) trichloroacetic acid (TCA), and then centrifuged at 18,000× *g* for 10 min. Then, 0.5 mL of 0.5% (*w/v*) TBA in 0.6% (*w/v*) TCA was added to 0.5 mL of the supernatant. The mixtures were heated at 95 °C for 30 min and quickly cooled in an ice bath to stop the reaction. After centrifugation at 10,000× *g* for 10 min, the absorbance of the supernatant at 440, 532, and 600 nm was recorded. The concentration of TBARS (nmol g^−1^ DW) was calculated by using the extinction coefficient of 155 mM^−1^ cm^−1^, and the results were expressed as nmol MDA equivalents per gram.

H_2_O_2_ was detected spectrophotometrically according to Velikova [142]. The supernatant was homogenized in 10% TCA (*w/v*) in an ice bath. After centrifugation at 12,000× *g* for 10 min, the 0.5 mL extraction solution was mixed with a 0.5 mL potassium phosphate buffer (pH 7.5) and 1 mL potassium iodide (1 M). The absorbance of the supernatant was read at 390 nm. The concentration of H_2_O_2_ was obtained using a standard curve.

### 4.7. Measurement of Antioxidant Enzymes

Superoxide dismutase (SOD, EC1.15.1.1): Frozen leaves and roots (0.5 g) were homogenized in 500 µL of 0.15 M Tris-HCl buffer (pH 7.5), containing 50 mg polyvinylpyrrolidone (PVP) on ice, and were then centrifuged twice at 14,000× *g* for 10 min at 4 °C. The supernatant was used for the SOD activity assay. The total SOD activity was determined by measuring the inhibition of the photochemical reduction of nitro blue tetrazolium (NBT), as described by Beyer and Fridovich [143]. One unit (1UI) of SOD activity was defined as the amount of enzymes that inhibited 50% of NBT photoreduction, monitored at 560 nm. The SOD activity value was measured in the unit min^−1^ mg protein^−1^.

Ascorbate peroxidase (APX, EC1.11.1.11): APX was assayed using the method described by Amako et al. [144]. To the enzyme extract (100 µL), 2.9 mL of the reaction mixture was added, containing 50 mM of the sodium phosphate buffer (pH 7.0) including 0.2 mM EDTA, 0.5 mM ascorbic acid, and 100 µM H_2_O_2_. The decrease in absorbance was recorded at 290 nm for 3 min. One enzyme unit was defined as µmol mg^−1^ protein oxidized ascorbate per min.

Peroxidase (POX, EC 1.11.1.7): POX was measured according to the method described by Polle et al. [145]. The reaction mixture contained 0.1 mL of the enzyme extract, 3 mL of 1 M phosphate buffer (pH 7.0), 20 mM of guaiacol, and 40 mM of H_2_O_2_. POX activity was determined at 470 nm by its ability to convert guaiacol to tetraguaiacol (ε = 26.6 mM^−1^ cm^−1^). One unit of POX activity was defined as an absorbance change of 0.01 unit min^−1^.

Polyphenol oxidase (PPO, EC1.14.18.1): PPO was estimated by the method used by Hori et al. [146]. The assay solution contained 20 mM of catechol in a phosphate buffer (0.1 M, pH 7). The reaction was started by adding 100 mL of the enzymatic extract. PPO activity was expressed in an enzyme unit per mg^−1^ protein. One unit of PPO activity was defined as the amount of enzyme causing an increase in the absorbance of 0.001 min^−1^ at 420 nm.

### 4.8. Soil Analysis

The physicochemical properties of agricultural field soil were evaluated after the experiment to assess the effect of vermicompost and/or AMF applied alone, or in combination, on soil fertility. Five homogeneous rhizospheric soil samples at a 0–20 cm depth were collected for each treatment applied. The samples were dried and sieved to measure their pH, electrical conductivity (EC), total organic matter (TOM), assimilable P, N, Ca, K, and Fe, and their glomalin contents. The pH and electrical conductivity (EC) were measured on a soil suspension diluted to 1/5 (*v/v*) using a pH meter (HI 9025) and a conductivity meter HI-9033 (Hanna Instruments, Padova, Italy), respectively. The determination of total organic matter was carried out according to the Aubert method [147]. The oxidation of organic matter was carried out by adding potassium dichromate in the presence of sulfuric acid. The assimilable phosphorus was determined according to Olsen and Sommers [148]. Before and after the harvest, K, Ca, N, and Fe were determined using portable X-ray fluorescence spectrometers (PXRF, Tracer III-SD; SN T3S2102; Bruker Elemental Kennewick, WA, USA).

The total glomalin-related soil protein (T-GRSP) and the easily extractable GRSP (EE-GRSP) were examined according to Cornejo et al. [149]. T-GRSP was extracted from soil (2 g) with 8 mL 50 mM of sodium citrate (pH 8.0), followed by autoclaving for 1 h at 121 °C. For EE-GRSP, the soil samples (2 g) were extracted with 8 mL 20 mM of sodium citrate (pH 7.0), followed by autoclaving for 30 min at 121 °C. For both fractions, the supernatants were separated by centrifugation at 10,000× *g* for 1 h. The T-GRSP extraction was carried out five times until the solution was straw-colored.

The protein content in the crude extracts was determined according to the Bradford method [140].

### 4.9. Statistical Analyses

Statistical analyses of the data were performed using SPSS version 23.0 statistical software (IBM, Armonk, NY, USA). The data were analyzed by an analysis of variance (ANOVA) to assess differences among treatments. The. mean separation was determined using Tukey’s honest significant difference test using a significance level of 5% (*p* ≤ 0.05).

In order to integrate all the data, a complete dataset comprising of soil analyses, growth, physiological data, and biochemical data of quinoa leaves and roots was subjected to a principal component analysis (PCA). Initially, index values for each treatment were calculated by assessing the response of drought stress compared to its control value. The responses of all the traits under each treatment were combined and used as index values for the PCA. These index values were used to identify the correlation of response variable vectors and treatments across the ordination space. The PCA was produced using XLSTAT v. 2016 (Addinsoft, NY, USA), and the heatmap was performed using the software GraphPad^®^ Prism v9.0 (GraphPad Software, San Diego, CA, USA). Percentage contributions of principal component (PC) variables are shown in Table S1.

The hierarchical cluster analysis (HCA) was executed on the correlation matrix of various growth, physiological, and biochemical characteristics of the quinoa plants under the two water regimes (DS and WW), illustrating the distinction between the variables and the different treatments applied. HCA was created using the R software.

The experiment data presented are mean values based on five replicates ± the standard error (SE) per treatment.

## 5. Conclusions

**Figure 6:**
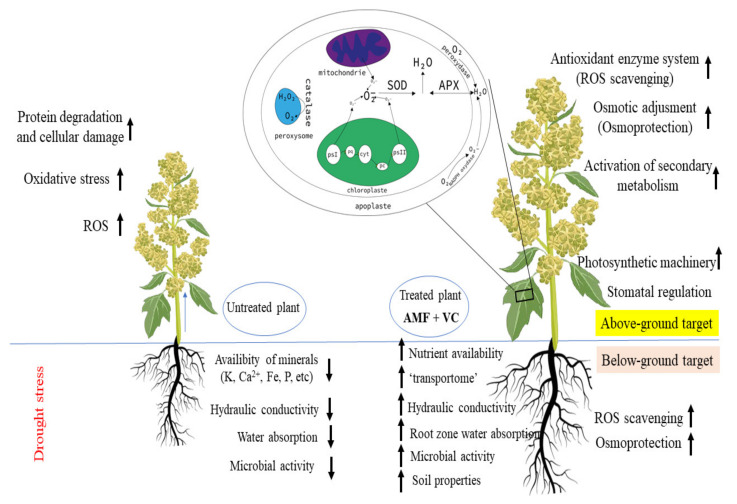
General overview of biostimulants’ (AMF+VC) underlying mechanisms to help field-grown quinoa cope with drought stress.

Taken together, these results demonstrate that bioinoculation, enriched with vermicompost, rather than applying them separately, is feasible for quinoa growing improvements without compromising the yield and soil nutrients under droughted conditions. More interestingly, this combination of AMF+VC, in turn, had positive consequences for the plant biomass, nutrition, and mycorrhizal colonization/microbial environment. Investigating the agro-physiological and biochemical mechanisms governing the drought-induced reshaping of the microbiome helped reveal how quinoa can sense stress and reshape their microbiome. Likely, these biological agents’ positive effects on plant biomass, nutrition, and tolerance were mediated by nutrient availability in the soil, ‘transportome’ in AM symbiosis, and physio-metabolic mechanisms underlying the tolerance of quinoa to drought. In a practical and experimental sense, mechanisms such as increasing soil fertility and stimulating of plant growth, as well as the physiological and biochemical attributes of quinoa, steadily improved the antioxidant enzyme defense, reduced the accumulation of the oxidative stress markers, and improved soil physicochemical properties (Figure 6). These findings also have implications for strategies to harness microbial communities to confer drought tolerance in field crops through identifying species that are effective at colonizing the endosphere for sustained protection. The increase in quinoa biomass and yield in the soil where the moisture was reduced may have important implications in the context of increased climate variability for a more sustainable agricultural practice.

## Figures and Tables

**Table 1:** Total soluble sugar (TSS), protein, hydrogen peroxide (H_2_O_2_), and malondialdehyde (MDA) of quinoa leaves and roots under well-watered (WW) and drought stress (DS) conditions after application of vermicompost (VC) and arbuscular mycorrhizal fungi (AMF) alone or in combination.

		TSS (mg g^−1^ DM)		Protein (mg g^−1^ DM)		H_2_O_2_ (nmol g^−1^ DM)		MDA (nmol g^−1^ DM)	
Treatments		Leaf	Root	Leaf	Root	Leaf	Root	Leaf	Root
WW	Control	149.4 ± 2.5 a	40.5 ± 4.0 e	44.9 ± 1.5 ab	9.9 ± 0.3 bc	20.3 ± 0.3 cd	33.5 ± 0.6 b	5.2 ± 0.2 bc	2.0 ± 0.05 c
	VC5	158.6 ± 1.9 a	46.8 ± 0.7 de	45.1 ± 1.3 ab	11.9 ± 0.2 a	18.0 ± 0.8 d–f	26.2 ± 1.2 cd	3.4 ± 0.3 cd	2.0 ± 0.04 c
	VC10	157.0 ± 1.7 a	75.7 ± 0.5 bc	49.3 ± 2.3 a	11.8 ± 0.2 a	15.3 ± 0.5 ef	6.7 ± 0.3 h	3.8 ± 1.1 c–d	1.0 ± 0.02 e
	AMF	149.0 ± 5.2 a	49.4 ± 1.3 de	49.8 ± 1.5 a	11.8 ± 0.3 a	15.0 ± 1.5 f	7.2 ± 0.4 h	2.2 ± 0.3 d	0.5 ± 0.02 f
	AMF+VC5	151.8 ± 6.2 a	42.5 ± 0.4 e	49.9 ± 2.8 a	11.6 ± 0.4 a	18.1 ± 0.5 de	20.3 ± 0.4 ef	2.6 ± 0.9 d	0.5 ± 0.04 f
	AMF+VC10	147.2 ± 2.2 a	53.1 ± 1.2 de	49.3 ± 1.5 a	11.4 ± 0.3 a	19.2 ± 1.7 d	14.6 ± 0.6 g	3.1 ± 0.9 d	0.3 ± 0.03 f
DS	Control	100.1 ± 3.5 d	53.9 ± 1.3 de	33.9 ± 1.7 c	7.5 ± 0.2 e	29.0 ± 1.8 a	43.8 ± 2.1 a	8.0 ± 0.5 a	3.5 ± 0.11 a
	VC5	103.6 ± 6.2 cd	72.0 ± 8.0 c	41.2 ± 2.0 b	8.2 ± 0.5 de	24.8 ± 0.7 b	41.8 ± 2.3 a	7.0 ± 0.8 ab	3.0 ± 0.1 b
	VC10	120.8 ± 4.5 b	88.0 ± 8.0 ab	42.8 ± 1.7 b	10.9 ± 0.9 ab	22.3 ± 0.7 bc	28.2 ± 0.8 c	6.4 ± 0.4 ab	2.0 ± 0.03 c
	AMF	111.1 ± 2.6 b–d	57.1 ± 1.3 d	42.4 ± 2.5 b	9.2 ± 0.5 cd	22.9 ± 1.3 bc	21.6 ± 1.5 d–f	6.0 ± 0.6 b	1.5 ± 0.02 d
	AMF+VC5	113.7 ± 5.8 bc	93.3 ± 9.2 a	39.5 ± 1.5 bc	9.2 ± 0.4 cd	24.3 ± 0.7 b	23.8 ± 0.5 c–e	6.6 ± 0.4 ab	2.0 ± 0.03 c
	AMF+VC10	116.7 ± 6.9 bc	99.5 ± 4.1 a	40.1 ± 1.3 b	11.2 ± 0.1 a	22.4 ± 0.3 bc	18.2 ± 3.8 fg	6.0 ± 0.8 b	1.3 ± 0.25 de

Values are means ± SE. The values of each column labelled by different letters indicate significant differences assessed by Tukey’s test (*p* < 0.05).

**Table 2:** Leaves and roots antioxidant enzyme (SOD, APX, POX, and PPO) activity of quinoa plants under well-watered (WW) and drought stress (DS) conditions after application of vermicompost (VC) and arbuscular mycorrhizal fungi (AMF) alone or in combination. APX: ascorbate peroxidase, EU: enzyme unit, POX: peroxidase, PPO: polyphenoloxidase, SOD: superoxide dismutase.

		SOD (EU mg^−1^ Protein)		APX (EU mg^−1^ Protein)		POX (EU mg^−1^ Protein)		PPO (EU mg^−1^ Protein)	
Treatments		Leaf	Root	Leaf	Root	Leaf	Root	Leaf	Root
WW	Control	10.71 ± 0.51 c	6.35 ± 0.69 a–c	0.94 ± 0.04 ef	0.60 ± 0.01 e	0.33 ± 0.01 b	1.04 ± 0.03 ef	0.50 ± 0.01 c–e	0.53 ± 0.02 g
	VC5	11.94 ± 1.32 a–c	5.40 ± 0.52 c	0.93 ± 0.01 ef	0.62 ± 0.05 de	0.32 ± 0.01 bc	0.93 ± 0.15 fg	0.48 ± 0.02 ef	0.76 ± 0.02 bc
	VC10	11.92 ± 0.74 a–c	7.41 ± 0.49 a	0.91 ± 0.04 ef	0.66 ± 0.02 b–e	0.30 ± 0.00 c	1.14 ± 0.06 e	0.46 ± 0.01 f	0.67 ± 0.05 d–f
	AMF	11.29 ± 0.77 bc	6.22 ± 0.13 a–c	0.89 ± 0.05 f	0.67 ± 0.01 b–e	0.29 ± 0.01 c	1.01 ± 0.06 ef	0.49 ± 0.02 d–f	0.61 ± 0.01 f
	AMF+VC5	11.95 ± 0.74 a–c	5.90 ± 0.65 bc	1.02 ± 0.03 de	0.66 ± 0.01 b–e	0.29 ± 0.01 c	1.43 ± 0.02 d	0.46 ± 0.01 f	0.65 ± 0.02 ef
	AMF+VC10	11.21 ± 0.80 bc	5.91 ± 0.67 bc	0.93 ± 0.01 ef	0.63 ± 0.01 c–e	0.29 ± 0.00 c	0.73 ± 0.06 g	0.46 ± 0.00 f	0.64 ± 0.00 ef
DS	Control	11.34 ± 1.13 bc	6.11 ± 0.56 a–c	1.08 ± 0.02 cd	0.64 ± 0.04 c–e	0.38 ± 0.01 a	1.71 ± 0.12 c	0.59 ± 0.01 a	0.65 ± 0.02 ef
	VC5	13.38 ± 0.43 ab	5.65 ± 0.36 bc	1.18 ± 0.06 bc	0.69 ± 0.03 a–d	0.35 ± 0.02 ab	1.78 ± 0.07 bc	0.57 ± 0.01 ab	0.93 ± 0.01 a
	VC10	13.88 ± 0.50 a	6.94 ± 0.34 ab	1.33 ± 0.06 a	0.73 ± 0.04 ab	0.34 ± 0.01 b	1.98 ± 0.02 ab	0.52 ± 0.01 cd	0.73 ± 0.01 b–d
	AMF	13.55 ± 0.64 ab	6.78 ± 0.53 a–c	1.18 ± 0.03 bc	0.76 ± 0.03 a	0.33 ± 0.01 b	2.07 ± 0.02 a	0.53 ± 0.02 bc	0.69 ± 0.02 c–e
	AMF+VC5	13.35 ± 0.80 ab	7.03 ± 0.14 ab	1.12 ± 0.07 cd	0.74 ± 0.01 ab	0.34 ± 0.02 b	1.70 ± 0.06 c	0.51 ± 0.00 c–e	0.78 ± 0.04 b
	AMF+VC10	13.22 ± 0.96 ab	7.34 ± 0.12 a	1.28 ± 0.05 ab	0.71 ± 0.05 a–c	0.33 ± 0.01 b	1.96 ± 0.05 ab	0.51 ± 0.01 c–e	0.73 ± 0.04 b–d

Values are means ± SE. The values of each column labelled by different letters indicate significant differences assessed by Tukey’s test (*p* < 0.05).

**Table 3:** Physicochemical parameters of post-harvest agricultural soil of quinoa plants grown under two water regimes (WW: well-watered and DS: drought stress) after application (or not; control) of vermicompost (VC) and arbuscular mycorrhizal fungi (AMF) alone or in combination.

		pH	EC (dS/m)	TOM (mg/g)	P (mg/kg)	K (mg/kg)	Ca (%)	Fe (%)	N (g/kg)	EE-GRSP (mg/g dry soil)	T-GRSP (mg/g dry soil)
WW	Control	8.2 ± 0.2 a	0.71 ± 0.02 bc	196.1 ± 4.4 c	29.2 ± 1.52 cd	5983 ± 58.3 a	1.1 ± 0.01 g	3.6 ± 0.02 ab	0.9 ± 0.04 g	1.7 ± 0.13 e	6.5 ± 0.40 bc
	VC5	7.9 ± 0.1 a	0.87 ± 0.06 a	234.3 ± 4.4 b	135.9 ± 69.53 b	5835 ± 56.4 a–c	1.2 ± 0.01 bc	3.4 ± 0.02 h	1.4 ± 0.09 d	2.6 ± 0.09 a	7.0 ± 1.00 a–c
	VC10	8.2 ± 0.2 a	0.69 ± 0.08 bc	259.8 ± 7.6 a	91.0 ± 1.65 bc	5428 ± 54.9 d	1.2 ± 0.01 ab	3.5 ± 0.02 de	1.9 ± 0.09 b	2.4 ± 0.14 ab	7.6 ± 0.13 ab
	AMF	8.2 ± 0.1 a	0.69 ± 0.02 bc	221.6 ± 7.6 b	77.8 ± 3.87 b-d	5969 ± 56.7 a	1.1 ± 0.01 ef	3.6 ± 0.02 c–e	0.7 ± 0.03 h	2.1 ± 0.05 cd	7.0 ± 0.70 a–c
	AMF+VC5	8.2 ± 0.0 a	0.69 ± 0.00 bc	244.5 ± 7.6 ab	112.3 ± 2.92 b	5693 ± 55.2 c	1.2 ± 0.01 de	3.5 ± 0.02 ef	1.1 ± 0.02 f	2.1 ± 0.08 cd	7.0 ± 0.16 a–c
	AMF+VC10	8.2 ± 0.0 a	0.79 ± 0.03 a–c	241.9 ± 6.2 ab	103.5 ± 3.13 b	5863 ± 55.7 ab	1.2 ± 0.01 a	3.4 ± 0.02 gh	1.7 ± 0.03 c	2.5 ± 0.11 a	7.8 ± 0.03 a
DS	Control	8.2 ± 0.2 a	0.67 ± 0.02 c	183.4 ± 7.6 c	18.7 ± 1.57 d	5699 ± 54.9 bc	1.2 ± 0.01 cd	3.7 ± 0.02 a	1.2 ± 0.01 ef	1.9 ± 0.07 de	3.1 ± 0.11 d
	VC5	8.2 ± 0.0 a	0.77 ± 0.01 a–c	241.9 ± 4.4 ab	210.9 ± 5.21 a	5763 ± 55.3 bc	1.2 ± 0.01 bc	3.5 ± 0.02 fg	1.7 ± 0.05 c	1.4 ± 0.07 f	7.7 ± 0.21 a
	VC10	8.3 ± 0.2 a	0.83 ± 0.08 ab	229.2 ± 4.4 b	240.3 ± 20.16 a	5497 ± 58.1 d	1.1 ± 0.01 g	3.6 ± 0.03 a–c	2.1 ± 0.05 a	2.1 ± 0.09 cd	7.6 ± 0.13 ab
	AMF	8.3 ± 0.2 a	0.74 ± 0.11 a–c	229.2 ± 4.4 b	134.8 ± 9.07 b	5186 ± 56.7 e	1.2 ± 0.01 d	3.6 ± 0.03 b–d	1.2 ± 0.02 ef	2.2 ± 0.09 bc	6.2 ± 0.13 c
	AMF+VC5	8.2 ± 0.2 a	0.68 ± 0.02 bc	231.8 ± 4.4 b	129.6 ± 2.59 b	5698 ± 59.0 c	1.2 ± 0.01 d	3.6 ± 0.03 b–e	1.3 ± 0.05 de	2.2 ± 0.09 bc	7.1 ± 0.31 a–c
	AMF+VC10	8.2 ± 0.2 a	0.80 ± 0.01 a–c	243.1 ± 22.5 ab	227.7 ± 7.10 a	4766 ± 49.5 f	1.1 ± 0.01 fg	3.4 ± 0.02 gh	1.4 ± 0.03 d	2.5 ± 0.09 a	7.3 ± 0.16 a–c

EC: electrical conductivity, TOM: total organic matter, C/N: carbon-to-nitrogen ratio, P: phosphorous, K: potassium, Ca: calcium, Fe: iron, EE-GRSP: easily extractable glomalin-related soil protein, T-GRSP: total glomalin-related soil protein.

**Table 4:** Initial analysis of the physicochemical parameters of agricultural soil and vermicompost (VC) used in this study.

	EC (dS m^−1^)	pH	TOM (mg/g)	P (mg/kg)	TKN (g/kg)	K (mg/kg)	Ca (%)	Fe (%)	C/N	Loam (%)	Clay (%)
AS	1.7 ± 0.6	7.9 ± 0.1	13 ± 3	31 ± 2	1.5 ± 0.1	636 ± 54.1	1.2 ± 0.1	3.75 ± 0.1	-	24 ± 1.6	52 ± 4.3
VC	1.5 ± 0.6	7.1 ± 0.6	610 ± 6	700 ± 0.6	27.0 ± 0.6	25 ± 0.6	45.0 ± 0.6	-	13.2 ± 0.6	-	-

AS: Agricultural soil; EC: electrical conductivity, TOM: total organic matter; P: phosphorus, TKN: total Kjeldahl nitrogen. K: potassium, Ca: calcium, Fe: iron, C/N: carbon-to-nitrogen ratio.

## Supplementary Materials

The supplementary material for this article can be found online at https://www.mdpi.com/article/10.3390/plants11030393/s1. Supplementary Table S1. Loading values and percent contribution of variables on the axis identified by the principal component analysis for all treatments under drought and well-watered conditions.
