# Supplementary material for: The Native Arbuscular Mycorrhizal Fungi and Vermicompost-Based Organic Amendments Enhance Soil Fertility, Growth Performance, and the Drought Stress Tolerance of Quinoa
Source: Plants (Basel). 2022 Jan 31;11(3):393. doi: 10.3390/plants11030393 (PMC8838481; doi:10.3390/plants11030393)
Supplement: Supplementary file 1 [file plants-11-00393-s001.zip › plants-1568489-supplementary.pdf]

Supplementary Table S1 Loading values and percent contribution of variables on the axis identified by the principal component analysis for all treatments under drought and well-watered conditions

Variable	**PC1**		**PC2**		**PC3**	
	Loading values	Contribution of variable (%)	Loading values	Contribution of variable (%)	Loading values	Contribution of variable (%)
SH	**-0.931**	4.334	-0.150	0.333	0.126	0.448
RL	**-0.808**	3.264	0.034	0.017	-0.077	0.167
SDW	**-0.920**	4.236	0.007	0.001	0.200	1.124
RDM	**-0.963**	4.642	-0.011	0.002	-0.020	0.011
SFW	**-0.951**	4.521	0.094	0.130	0.144	0.581
Ψ_{leaf}	**-0.940**	4.426	0.201	0.599	-0.007	0.001
g_s	**-0.955**	4.564	0.158	0.371	0.109	0.331
F_v/F_m	**-0.854**	3.647	0.349	1.815	0.036	0.037
Chl *a*	**-0.869**	3.777	0.082	0.100	0.098	0.270
Chl *b*	**-0.938**	4.404	-0.025	0.009	-0.133	0.499
Chl *T*	**-0.955**	4.565	0.009	0.001	-0.059	0.098
Caro	**-0.902**	4.067	0.096	0.137	0.092	0.237
Prot L	**-0.962**	4.627	0.109	0.178	0.074	0.152
Prot R	**-0.829**	3.442	0.338	1.700	0.055	0.086
TSS L	**-0.936**	4.388	-0.155	0.356	-0.017	0.008
TSS R	0.491	1.206	**0.697**	7.209	0.241	1.625
H_2O_2 L	**0.953**	4.547	-0.121	0.217	-0.130	0.476
H_2O_2 R	**0.725**	2.627	-0.425	2.682	-0.407	4.632
MDA L	**0.965**	4.658	-0.053	0.042	0.063	0.111
MDA R	-0.335	0.562	**-0.760**	8.591	-0.308	2.654
POX L	**0.922**	4.256	-0.282	1.183	-0.106	0.316
POX R	**0.809**	3.271	0.392	2.278	0.211	1.250
PPO L	**0.891**	3.970	-0.224	0.747	-0.082	0.188
PPO R	0.432	0.933	**0.504**	3.782	**-0.531**	7.914
SOD L	0.516	1.334	**0.744**	8.234	0.013	0.004
SOD R	0.209	0.218	0.418	2.601	0.803	18.073
APX L	**0.747**	2.792	**0.527**	4.123	0.088	0.216
APX R	0.455	1.038	**0.706**	7.415	0.318	2.835
pH	0.156	0.122	0.163	0.393	**0.612**	10.507
EC	0.027	0.004	0.480	3.430	**-0.616**	10.622
TOM	-0.389	0.757	**0.790**	9.281	-0.266	1.981
P	0.266	0.353	**0.856**	10.889	-0.264	1.952
K	-0.412	0.850	**-0.657**	6.406	-0.121	0.407
Ca	-0.248	0.307	-0.043	0.027	**-0.610**	10.417
Fe	0.412	0.849	-0.465	3.207	**0.645**	11.651
N	0.177	0.157	0.442	2.902	**-0.528**	7.803
EE-GRSP	-0.376	0.706	0.417	2.580	-0.082	0.188
T-GRSP	**-0.562**	1.581	**0.637**	6.032	-0.068	0.129
Eigenvalue		19.985		6.729		3.568
Variability (%)		52.593		17.708		9.389
Cumulative %		52.593		70.301		79.689

SH: shoot height; RL: root length; SDW: shoot dry weight; RDM: root dry matter; SFW: seeds fresh weight; Ψ_{Leaf}: Leaf water potential; g_s: stomatal conductance; F_v/F_m: chlorophyll fluorescence; Chl *a*: chlorophyll a; Chl *b*: chlorophyll b; Chl *T*: total chlorophyll; Car: Carotenoid; Prot L: Protein in leaf; Prot R: Protein in the root; TSS L: total soluble sugar in leaf; TSS R: total soluble sugar in the root; H_2O_2 L: hydrogen peroxide in leaves; H_2O_2 R: hydrogen peroxide in roots; MDA L: malondialdehyde in leaf; MDA R: malondialdehyde in the root; POX L: peroxidase activity in leaves; POX R: peroxidase activity in the root; PPO L: polyphenol oxidase activity in leaf; PPO R: polyphenol oxidase activity in the root; SOD L: superoxide dismutase activity in leaf; SOD R: superoxide dismutase activity in root; APX L: ascorbate peroxidase in leaf; APX R: ascorbate peroxidase in the root; EC: electrical conductivity; TOM: total organic matter; P: phosphorus; K: potassium; Ca: Calcium; Fe: iron; N: nitrogen; EE-GRSP, Easily extractable glomalin-related soil protein; T-GRSP, total glomalin-related soil;
